# Comparison of training and match load between metabolic and running speed metrics of professional Spanish soccer players by playing position

**DOI:** 10.5114/biolsport.2022.110884

**Published:** 2021-11-20

**Authors:** Berni Guerrero-Calderón, José Alfonso Morcillo, Marcos Chena, Alfonso Castillo-Rodríguez

**Affiliations:** 1 Department of Physical Education and Sports. Faculty of Sport Sciences. University of Granada, Granada, Spain; 2 Department of Physical Education and Sports. University of Jaen, Jaen, Spain; 3 Strength & Conditioning Coach Real Racing Club Santander, Spain

**Keywords:** Physical responses, Time motion analysis, Metabolic power, GPS, Competition, Performance

## Abstract

The aims of this study were to compare the training and match load of professional soccer players according to the playing position, and analyse the relationship between the metabolic and running speed metrics. Thirty professional male soccer players belonging to a Spanish First Division team were analysed using global positioning system devices (GPEXE Pro 18.18 Hz) during training and competition (*n* = 36 training weeks and *n* = 41 matches). The results showed significant differences between positions on match day; central midfielders covered higher total distance and low- and medium-speed running distance (moderate to large effect size) than central defenders, external defenders and forwards; forwards performed more metabolic power events than central defenders, central midfielders and wide midfielders; and central defenders showed the lowest very-high-speed running. Different patterns were observed in training. Furthermore, the equivalent-distance index showed a strong correlation with accelerations and decelerations events. The main findings were that the physical responses found in training did not correspond with match demands by position; both metabolic and traditional approaches should be used together for load monitoring in professional soccer players; and finally, metabolic power events and the equivalent-distance index seem to be variables that help to differentiate more clearly the characteristics of the player, taking into account their playing position.

## INTRODUCTION

High-level soccer competition is highly physically demanding for players, and monitoring the training load (TL) is paramount to attain appropriates training programmes and load programming in order to optimize the physical capacity of players and reduce the risk of injury [1–3]. Professional players accumulate a large number of matches throughout the season and inadequate loads may lead to an increased injury risk [4, 5]. With the exception of preseason and resting periods, in a team sport such as soccer, where players compete once or twice per week and show large load variability between training days (TDs) and positions [6–8], the load periodization is commonly developed by weekly microcycles based on match day (MD), which usually coincides on weekends [9, 10]. Considering that players train to compete, it is logical to programme the TL relative to match demands.

It is imperative to monitor and programme TL in order to withstand the specific competition demands and requirements according to position. High-level soccer competition is increasingly demanding and players must prepare for a specific match scenario [6, 11]. Most studies analyse time-motion variables based on running speed or intensity thresholds such as distances covered at different speed ranges, high-intensity events or the number of acceleration and deceleration events for monitoring the training and match load [5, 9, 12]. However, although this traditional running speed-based approach is considered valid for load monitoring, several authors argue that it does not represent the real physical demands in soccer as it not consider the accelerative phases in high-intensity running and support the metabolic approach as more accurate method to obtain the energy cost derived from the locomotion activity such as accelerations and decelerations or distances covered at different speeds per unit of time of team athletes [13–15]. These metabolic variables represent the athlete’s aerobic capacity and measure the energy supply [16], thus allowing an accurate estimate of energy cost and metabolic demands when several locomotion activities are considered together [17]. For instance, the equivalent distance index (EDI) can reflect the intermittent nature of soccer as it represents the percentage of distance that the player would have covered at a steady pace, using the total energy consumed throughout the match [16]. In addition, Castagna et al. [15] found positive relationships between metabolic and running speed metrics, thus supporting the suitability of this approach to represent high-speed activity.

However, there is still scarce research analysing the metabolic approach variables (e.g., average metabolic power, maximum power or power events) in training- and match-load monitoring by playing position and their relationship with running speed-based metrics.

To the authors’ knowledge, variables from both approaches should be considered together in load monitoring to improve the understanding of physical demands and thus improve load programming throughout the microcycle. Although there may be a good correlation between the two approaches, we assume that there are different physical demands according to position and TL should be programmed to satisfy the competition demands [15]. The authors hypothesize that the load performed in the different training sessions does not meet the competitive demands per position, suggesting inadequate TL programming. Therefore, the current investigation has two aims: (1) to compare the metabolic and traditional running speed-based approaches both in training and competition by playing position, and (2) analyse the relationship between metabolic and running speed metrics in professional soccer players belonging to a Spanish First Division team. These results may inform about the positions that perform inadequate loads on TDs regarding MD, and also the specific metrics to take into account for load monitoring.

## MATERIALS AND METHODS

### Subjects

Thirty professional male soccer players (22.8 ± 0.8 years; 177.8 ± 6.9 cm; 73.3 ± 5.7 kg) belonging to a Spanish First Division team during the season 2015–2016 participated in this study. Players were classified by playing position [9, 10, 18]: central defender (CD), external defender (ED), central midfielder (CM), wide midfielder (WM) and forward (FO). The maximum oxygen uptake (VO_2_) of these playing positions were 6.59 ± 0.95 l·min^-1^; 6.61 ± 0.92 l·min^-1^; 7.22 ± 1.05 l·min^-1^; 6.91 ± 1.03 l·min^-1^; and 6.88 ± 0.99 l·min^-1^, respectively. Goalkeepers were also excluded from the analysis. This study was approved by the Ethics Committee of the University of Granada (Number 471/CEIH/2018) and the club provided written informed consent.

### Instruments

All records were collected using wearable GPS technology (GPEXE Pro 18.18 Hz, GPEXE, Udine, Italy). GPS devices are valid tools for monitoring the locomotion responses of high-level soccer players both in training and matches [5, 9, 12]. The players were fitted with these devices 15 min before activity to avoid possible errors or delays with GPS’ activations. The number of satellites during the training and matches was 8 ± 1.

### Procedures

Training and match load data corresponding to the competitive period were recorded over the season. Records from preseason and the non-competition period (i.e., Christmas) were excluded to avoid variability in training records. In addition, training records for the twogame weeks, exclusively tactical sessions and records of players who did not complete the full training session were excluded from the analysis. For match analysis, only the records of players who played > 80 minutes were considered. Finally, a total of 33 training weeks and 38 matches were analysed. The data were grouped according to the number of days before the match day (MD): 4 days before the match (MD-4), 3 days before the match (MD-3), 2 days before the match (MD-2) and 1 day before the match (MD-1). Training data obtained before MD-4 were not considered for the analysis as these sessions do not involve any specific and representative content and are usually oriented to recovery [9, 19].

### Variables

Traditional running speed-based and metabolic metrics were analysed for this study. All these variables have been reported directly by GPS devices. The running speed metrics are as follows: number of acceleration events (ACC), considered as the number of speed increments equal to or greater than 2 m·s^-2^ during an interval time equal to or less than 0.5 seconds; number of deceleration events (DEC), number of braking or speed decrements ≤ -2 m·s^-2^ during an interval time ≤ 0.5 seconds; total distance covered (total distance); low-speed running distance (LSRD), i.e., distance covered at speeds < 14 km·h^-1^; medium-speed running distance (MSRD), from 14 to 18 km·h^-1^; high-speed running distance (HSRD), from 18 to 21 km·h^-1^; very-high-speed running distance (VHSRD), from 21 to 24 km·h^-1^; and sprint running distance (SPD), > 24 km·h^-1^. All the distances were recorded in metres. On the other hand, the metabolic metrics provide an estimation of the energy cost of accelerations and decelerations during intermittent activity of soccer and are as follows: averaged metabolic power (MP, on W·kg^-1^), i.e. energy used per unit of time; average metabolic power from actions developed at high intensity ≥ 20 W·kg^-1^ (MPev, on W·kg^-1^); number of high-intensity events (power events); maximum power (Pmax, in W·kg^-1^), peak energy reached during the activity; and the equivalent distance index (EDI, as a percentage), the percentage of distance that the player would have run at a constant pace using the total energy expended during the match. This metric is evaluated through the equation: ED (m) = energy expenditure (J·kg^-1^) / energy cost (3.6 J·kg^-1^·m^-1^) [20].

### Statistical analysis

Microsoft Excel 2016 (Microsoft Corp, Redmond, Washington, USA) and IBM SPSS Statistics for Mac, Version 25.0 (IBM Corp, Armonk, NY, USA) were used. The Kolmogorov-Smirnov test revealed that almost none of the data presented a normal distribution. Only MP and power events on MD-4; ACC, MSRD and MPev on MD-3; DEC, total distance, MSRD, EDI and Pmax on MD-2; and MP on MD-1 showed a normal distribution. Factorial analysis of variance tests were conducted for non-normal data (Kruskal-Wallis) and normal data (ANOVA) to explore the differences between positions among training days. Post-hoc analyses were performed with the Mann-Whitney U-test and Bonferroni test, respectively. Dunnett’s T3 test was applied (MSRD and MPev on MD-3) when the parametric data presented non-homogeneous variances. Effect size was calculated to determine the meaningfulness of the difference. The eta-squared (*η*^2^) has been shown to be an appropriate way to calculate the effect size for parametric and non-parametric variables [21]. The threshold values for effect size were as follows: 0.10 for small effects, 0.25 for moderate effects, and 0.40 for large effects [22]. To determine the within-subject variability of dependent variables, the coefficient of variation (CV) was calculated for each position among training days. Finally, a correlational analysis (Spearman’s coefficient test) was conducted for the motion activities and metabolic responses. The magnitude of the correlation was interpreted as follows: < 0.1, trivial; from 0.1 to 0.3, small; from 0.3 to 0.5, moderate; from 0.5 to 0.7, large; from 0.7 to 0.9, very large; and from 0.9 to 1.0, almost perfect [23]. The level of significance was set at *p* ≤ .05.

## RESULTS

### Traditional running speed-based approach

Table 1 shows the comparative data of traditional running speed-based metrics between microcycle days by playing position. Generally, significant differences were found both in competition and training. All metrics showed lower measures in training than MD. In MD, positional differences showed that FOs performed greater ACCs and DECs than CDs and EDs. CMs showed higher DEC (104.4 ± 25.9 events; *η*^2^ = .34) than CDs, EDs and WMs; higher ACCs than CDs and EDs; greater total distance than CDs, EDs and FOs (10760.0 ± 775.2 vs 8939.4 ± 1259.2, 9050.7 ± 642.1 and 9182.5 ± 1953.5 m, respectively; *η*^2^ = .39); and greater MSRD and HSRD (1572.9 ± 304.0 m, *η*^2^ = .46; and 543.2 ± 126.8 m, respectively; *η*^2^ = .39) than CDs and EDs. CDs and CMs covered the shortest SPD (108.9 ± 62.9 and 163.7 ± 128.3 m, respectively; *η*^2^ = .21), and CDs also showed the lowest VHSRD (161.5 ± 61.8 m; *η*^2^ = .26).

**TABLE 1:** Comparative data (mean ± SD) and eta-squared (*h*^2^) results of locomotion activities based on running speed between microcycle days by playing position. Sample size: CD (N = 89), ED (N = 61), CM (N = 71), WM (N = 76) and FO (N = 36).

Var.	Position	MD-4	MD-3	MD-2	MD-1	MD	p	h^2^
ACC	CD	49 ± 17.8	42.4 ± 13.8	38 ± 14.9	32 ± 8.2^e^	68.5 ± 13.0^c,e^	.08	.01
	ED	45.9 ± 17.2	41.9 ± 12.5	36.1 ± 13.2	31.2 ± 8.5^e^	63.4 ± 11.3^c,e^	.00	.04
	CM	42.7 ± 19.7	39.5 ± 14^d,e^	33.7 ± 16.6^e^	30.6 ± 11.1^e^	81.9 ± 21.2^a,b^	.03	.03
	WM	47.6 ± 18.6	45.1 ± 17.1^c^	40.1 ± 16.2	32.2 ± 10.8^e^	68.7 ± 16^c^	.00	.07
	FO	51.3 ± 21.9	48.8 ± 14.9^c^	43.1 ± 14.1^c^	41.1 ± 10.9^+^	82.2 ± 16.5^a,b^	.00	.16
DEC	CD	44.1 ± 18.6	39.6 ± 13.2^e^	34.3 ± 14.2	29.2 ± 10.5^e^	70.6 ± 15.7^c,d,e^	.20	.01
	ED	44.3 ± 18.5	41.8 ± 12.3^e^	36.3 ± 14.8	29.6 ± 8.9^e^	75.3 ± 11^c,e^	.00	.03
	CM	42.8 ± 19.1	39.7 ± 15.4^e^	36 ± 17.2	30.4 ± 11.9^e^	104.4 ± 25.9^a,b,d^	.12	.03
	WM	42.7 ± 16.7	41.9 ± 15.3^e^	36.6 ± 14.7	28.8 ± 9.8^e^	81.8 ± 14.5^a,c^	.00	.05
	FO	51.2 ± 23.8	49.1 ± 16.1^+^	43.9 ± 13.7	37.4 ± 10.2^+^	95.8 ± 22^a,b^	.00	.34
Total Distance (m)	CD	5136.4 ± 1086.7	4428.9 ± 1125.8	3763.3 ± 899.8	3351.2 ± 736.7	8939.4 ± 1259.2^c,d^	.20	.01
	ED	5017.8 ± 1142.1	4548.5 ± 983.8	3801.2 ± 945.6	3315.9 ± 733.3	9050.7 ± 642.1^c,d^	.25	.00
	CM	5306.5 ± 1163.3	4687.7 ± 1375.2	3973.4 ± 948	3611.7 ± 798.7	10760 ± 775.2^a,b,e^	.67	.01
	WM	5112.7 ± 1302.5	4628.2 ± 1091	3970.3 ± 911	3412.5 ± 749.9	9857.6 ± 1688.2^a,b^	.12	.01
	FO	5060.7 ± 1257.4	4467.7 ± 1217.9	3844.9 ± 826.3	3474.2 ± 751.7	9182.5 ± 1953.5^c^	.00	.39
LSRD (m)	CD	4141.6 ± 1061.6	3726.8 ± 920.9	3266.2 ± 715.7	2919.9 ± 623.7	7445.7 ± 1039.9^c^	.21	.01
	ED	3968.7 ± 1078.5	3702.6 ± 798	3202.9 ± 746.8	2832.5 ± 560.8	7322.1 ± 469.7^c^	.15	.01
	CM	4195.5 ± 1248.6	3865.5 ± 1073.2	3412.4 ± 717.5	3108.2 ± 634.4	8227.7 ± 460.1^+^	.73	.01
	WM	4018.3 ± 1142.3	3707.5 ± 863.7	3317.4 ± 688.6	2846.7 ± 577.1	7619.2 ± 1209.7^c^	.05	.02
MSRD (m)	FO	3872.3 ± 1173.9	3596.4 ± 948.8	3183.2 ± 637	2866.1 ± 559.1	6940.8 ± 1376.8^c^	.00	.28
	CD	705.8 ± 785.5	417.9 ± 172^c,d^	335 ± 158.9	287 ± 101.4^c,e^	896.7 ± 187.9^c,d^	.02	.02
	ED	681.4 ± 857.8^c^	442.6 ± 149.4^c^	350.4 ± 170.3	278.8 ± 108.8^c,e^	913.7 ± 177^c,d^	.00	.05
	CM	789.1 ± 787.6^b^	535.7 ± 251.9^a,b^	403.8 ± 180.7	357.4 ± 147.8^a,b^	1572.9 ± 304^a,b,e^	.09	.04
	WM	700.9 ± 685	494.5 ± 160.8^a^	394.9 ± 157.5	319.9 ± 120.8	1270.4 ± 373.8^a,b,c^	.00	.04
	FO	763.2 ± 776.2	484.7 ± 179.1	412.2 ± 137.6	345 ± 116.5^a,b^	1029 ± 237.1^c^	.00	.46
HSRD (m)	CD	182.6 ± 86.7^e^	166 ± 120.3^b,d,e^	104.5 ± 69.8^d^	90.3 ± 45.1^d,e^	326.4 ± 88.2^c,d,e^	.01	.02
	ED	205.4 ± 99.7	201.8 ± 97.2^a^	138.0 ± 78.8	106.7 ± 53.3^e^	381.8 ± 95.6^c,d^	.00	.05
	CM	230.7 ± 235.2	186.6 ± 130.3^d^	111.5 ± 75.8	102.4 ± 56.3^d,e^	543.2 ± 126.8^a,b^	.01	.05
	WM	231.9 ± 115.3	220.9 ± 100^a,c^	144.8 ± 76.5^a^	134.6 ± 78.3^a,c^	508.9 ± 159.5^a,b^	.00	.09
	FO	271.5 ± 197.8^a^	213.5 ± 103.6^a^	149.3 ± 76.3	146.7 ± 64.9^a,d,c^	498.5 ± 144.2^a^	.00	.39
VHSRD (m)	CD	73.6 ± 58.2^b,d^	81.5 ± 80.5^b,d,e^	41.6 ± 38.2^b,d,e^	39.9 ± 34.1^b,d,e^	161.5 ± 61.8^x^	.00	.06
	ED	104.3 ± 66.4^a,c^	121.8 ± 85.2^a,c^	69.3 ± 51.7^a,c^	60.5 ± 42.4^a,c^	234.6 ± 84.1^a^	.00	.09
	CM	67.9 ± 60.5^b,d^	77.7 ± 75.8^b,d,e^	36.8 ± 31.8^b,d,e^	34.9 ± 28^b,d,e^	252.4 ± 98.4^a^	.00	.12
	WM	100.9 ± 66.9^a,c^	130.8 ± 99.3^a,c^	75.4 ± 51.6^a,c^	70.9 ± 54.8^a,c^	252.8 ± 95.6^a^	.00	.13
	FO	98.7 ± 70.6	112.5 ± 86.5^a,c^	70.7 ± 45.9^c^	78.9 ± 58.2^a,c^	336.8 ± 136.5^a^	.00	.26
SPD (m)	CD	32.9 ± 47.2^b,d^	36.9 ± 53.8^b,d,e^	16.2 ± 22.6^b,d,e^	14.2 ± 20.2^b,d,e^	108.9 ± 62.9^b,d,e^	.00	.08
	ED	58.1 ± 55.7^a,c^	79.8 ± 66.6^a,c^	40.7 ± 36.9^a,c^	37.5 ± 44.2^a,c^	198.5 ± 87.1^a,c^	.00	.16
	CM	23.4 ± 32.3^b,d^	22.3 ± 29.3^b,d,e^	9.1 ± 12.8^b,d,e^	8.8 ± 12.7^b,d,e^	163.7 ± 128.3^b,d,e^	.00	.17
	WM	60.9 ± 66.9^a,c^	74.7 ± 75^a,c^	37.8 ± 35.4^a,c^	40.4 ± 64.1^a,c^	206.3 ± 147.7^a,c^	.00	.11
	FO	55.1 ± 65.1	60.8 ± 67.3^a,c^	29.6 ± 24.6^a,c^	37.6 ± 43.7^a,c^	377.4 ± 215^a,c^	.00	.21

Note: Var., variable; ACC, acceleration events; DEC, deceleration events; LSRD (< 14 km·h^-1^); MSRD, (14 to 18 km·h^-1^); HSRD (18 to 21 km·h^-1^); VHSRD (21 to 24 km·h^-1^); SPD (> 24 km·h^-1^); +, significantly higher than all other variables; x, significantly lower than all other variables;

a , statically significant difference with CD;

b , statically significant difference with ED;

c , statically significant difference with CM;

d , statically significant difference with WM;

e , statically significant difference with FO.

**FIG. 1:**
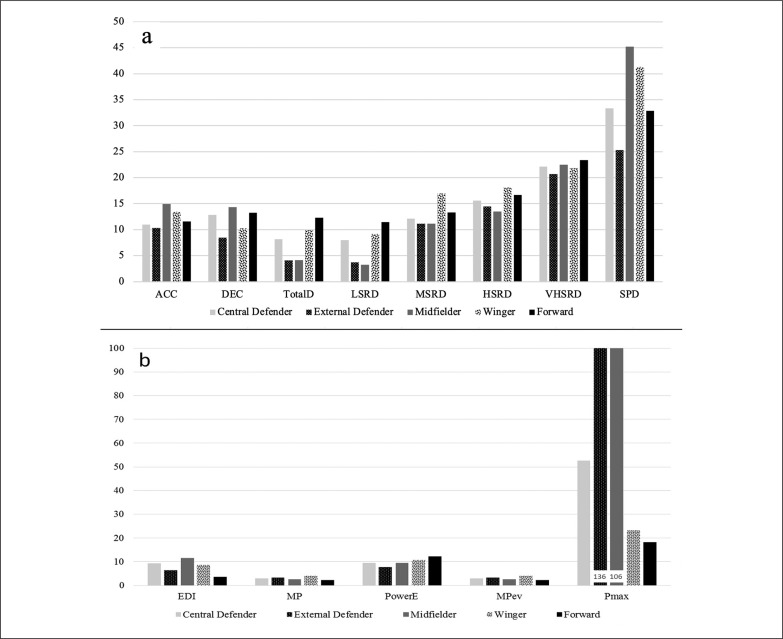
Coefficient of variation (CV) for running speed metrics (a) and metabolic variables (b) on match day by position. Note: Pmax values of ED and CM (b) are represented with a data label as they were higher than 100%

In MD-1, FOs showed the highest ACCs (41.1 ± 10.9 events) and DECs (37.4 ± 10.2 events); and greater HSRD (134.6 ± 78.3 m; *η*^2^ = .09) than CDs, EDs and CMs. CDs and CMs showed the shortest SPD again (40.4 ± 64.1 vs 14.2 ± 20.2 and 8.8 ± 12.7 m respectively; *η*^2^ = .11). FOs also performed the largest number of DEC on MD-3 (49.1 ± 16.1 events). On the other hand, when the CV was considered, different trends were found in MD by position (Figure 1a). The higher the running speed, the higher were the CVs.

### Metabolic approach

Table 2 shows the comparative data between microcycle days and positions considering the metabolic approach. Scarce differences were found between positions in MD; CDs and EDs performed the lowest number of power events (140.4 ± 23.0 138.7 ± 18.6 eve nts, respectively; *η*^2^ = .51); and lower MP (8.3 ± 0.6 8.4 ± 0.6 W·kg^-1^, respectively) than CMs and WMs (10.0 ± 0.7 and 9.3 ± 0.9 W·kg^-1^, respectively) and showed a large effect size (*η*^2^ = .45). However, CDs showed lower Pmax and MPev than EDs. In training, results showed similar variability between positions among TDs; CMs performed lower MPev and Pmax than EDs, WMs and FOs all TDs; and CDs showed lower MPev than EDs and FOs. In addition, FOs showed the highest EDI on MD-3 (14.0 ± 2.1%) and MD-1 (14.2 ± 1.6%). On the other hand, CVs showed very different trends on MD between positions in Pmax; EDs and CMs obtained the greatest values (136 and 106%, respectively), and the lowest CV was found in FOs (18%) (Figure 1b).

**TABLE 2:** Comparative data (mean ± SD) and eta-squared (*h*^2^) results of metabolic variables between microcycle days by playing position. Sample size: CD (N = 89), ED (N = 61), CM (N = 71), WM (N = 76) and FO (N = 36).

Var.	Position	MD-4	MD-3	MD-2	MD-1	MD	p	h^2^
EDI (%)	CD	12 ± 3^e^	12.6 ± 2.5^e^	12.5 ± 2.2^e^	12.2 ± 2^e^	11.9 ± 1.9^c,e^	.00	.04
	ED	12.4 ± 3.3	12.9 ± 2.2^e^	12.8 ± 2.2	12.6 ± 1.8^e^	12.5 ± 1.4^e^	.00	.06
	CM	11.4 ± 3.2^e^	12.2 ± 2.4^e^	12 ± 1.9^e^	11.8 ± 1.8^e^	13.3 ± 2.6^a^	.00	.07
	WM	12.1 ± 3.3	12.8 ± 2.7^e^	12.8 ± 2.5	12.2 ± 2^e^	12.2 ± 1.8^e^	.00	.12
	FO	13.9 ± 3.9^a,c^	14.0 ± 2.1^+^	14.1 ± 1.7^a,c^	14.2 ± 1.6^+^	14.4 ± 0.9^a,b,d^	.00	.18
Pmax (W · kg^-1^)	CD	82.5 ± 10.9^b^	83 ± 10.5^b,d,e^	81.6 ± 13.2^e^	82.1 ± 11.2^b,d,e^	117.1 ± 106.7^b^	.00	.07
	ED	89.8 ± 15^a,c^	90.8 ± 12.6^a,c^	86.8 ± 13.1^c^	92.5 ± 15.4^a,c^	183.4 ± 430.7^a^	.00	.12
	CM	80.8 ± 18^b,d,e^	79.5 ± 11.7^b,d,e^	76.6 ± 9.7^b,d,e^	81 ± 10.8^b,d,e^	155.9 ± 285	.00	.12
	WM	85.6 ± 10.9^c^	87.7 ± 13^a,c^	85.9 ± 12.1^c^	87.8 ± 12.5^a,c^	117.4 ± 47.1	.00	.12
	FO	87.9 ± 14.8^c^	92.5 ± 14.5^a,c^	90 ± 10.4^a,c^	93 ± 12.1^a,c^	120.1 ± 37.9	.01	.05
MP (W · kg^-1^)	CD	6.8 ± 1	6.6 ± 1.1^c^	6.5 ± 1.1	5.7 ± 1.1	8.3 ± 0.6^c,d^	.16	.02
	ED	6.7 ± 0.9	6.6 ± 1	6.6 ± 1.4	5.7 ± 1.1	8.4 ± 0.6^c,d^	.02	.02
	CM	7 ± 1	6.9 ± 1.3^a^	7.1 ± 1.3	6.1 ± 1.1	10 ± 0.7^a,b^	.18	.01
	WM	6.8 ± 1	6.9 ± 1	6.7 ± 1.3	5.9 ± 1.1	9.3 ± 0.9^a,b^	.05	.02
	FO	6.9 ± 0.9	6.7 ± 1.2	6.8 ± 1.1	6.1 ± 1.1	8.9 ± 0.8	.00	.45
Power Events	CD	90.8 ± 23.7	76.4 ± 24.1^e^	67.4 ± 25.6	54.9 ± 15^e^	140.4 ± 23^c,d,e^	.50	.01
	ED	86.1 ± 27	77.3 ± 22.5	66 ± 26.5	53.5 ± 17.1^e^	138.7 ± 18.6^c,d,e^	.01	.02
	CM	90.6 ± 24.1	79.5 ± 27.4	68.4 ± 28	57.8 ± 18.2	208.2 ± 34.2^a,b^	.15	.01
	WM	90.9 ± 26.8	84.2 ± 25	74.4 ± 26.5	57.1 ± 16.7	183.1 ± 33.9^a,b^	.01	.03
	FO	95.5 ± 32	86.3 ± 26.7^a^	77.9 ± 23.6	69.4 ± 17.3^a,b^	170.2 ± 36.1^a,b^	.00	.51
MPev (W · kg^-1^)	CD	27.5 ± 1.6^b,e^	28.1 ± 1.3^b,d,e^	27.5 ± 1.4^b,e^	28.3 ± 1.5^b,e^	28.8 ± 1.4^b,e^	.00	.11
	ED	28.6 ± 2.2^a,c^	29.5 ± 1.8^a,c^	28.7 ± 1.8^a,c^	29.6 ± 1.9^a,c^	30.4 ± 1.7^a,c,d^	.00	.15
	CM	27.2 ± 1.5^b,d,e^	27.7 ± 1.5^b,d,e^	27.1 ± 1^b,d,e^	27.7 ± 1.4^b,d,e^	28.6 ± 1.3^b,e^	.00	.15
	WM	28.1 ± 2.2^c^	28.9 ± 2^a,c^	28.2 ± 1.8^c^	28.9 ± 2^c,e^	28.2 ± 1.9^b,e^	.00	.17
	FO	28.6 ± 2^a,c^	29.4 ± 1.4^a,c^	28.5 ± 1^a,c^	30 ± 1.4^a,c,d^	30.7 ± 1.2^a,c,d^	.00	.27

Note: Var., variable; EDI, Equivalent Distance Index; Pmax, maximum power; MP, averaged Metabolic Power; MPev, Metabolic Power events average power; +, significantly higher than all other variables; x, significantly lower than all other variables;

a , statically significant difference with CD;

b , statically significant difference with ED;

c , statically significant difference with CM;

d , statically significant difference with WM;

e , statically significant difference with FO.

### Relationship between metabolic and traditional running speed-based variables

Spearman’s correlation analysis between the traditional running speed-based parameters and the new metabolic variables is shown in Table 3. Except EDI with LSRD and MSRD, and MPev with TotalD, LSRD and MSRD, where no correlations were found, all metabolic measures showed trivial to very large correlations with locomotion metrics; and very large correlations were found between power events and ACC, DEC, TotalD, LSRD, MSRD and HSRD.

**TABLE 3:** Spearman’s coefficient test of locomotion parameters and metabolic variables.

Variable	ACC	DEC	TotalD	LSRD	MSRD	HSRD	VHSRD	SPD
EDI	.534^**^	.490^**^	-.050^*^	-.038	-.009	.111^**^	.236^**^	.247^**^
Pmax	.496^**^	.449^**^	.230^**^	.241^**^	.211^**^	.316^**^	.452^**^	.474^**^
MP	.502^**^	.579^**^	.694^**^	.614^**^	.742^**^	.654^**^	.519^**^	.402^**^
Power Ev	.836^**^	.844^**^	.861^**^	.820^**^	.859^**^	.757^**^	.649^**^	.509^**^
MPev	.329^**^	.260^**^	.028	.016	.034	.311^**^	.530^**^	.521^**^

Note:

* (*p* ≤ .05),

** (*p* ≤ .01)

## DISCUSSION

This study aimed to analyse and compare the training and match load from both the metabolic and running speed-based approaches of high-level soccer players by playing position. The main findings were: 1) different physical responses were found in both training and match according to position; 2) CM was one of the most physically demanding positions in competition (large values of total distance, total high-intensity distance, ACCs and DECs). However, CM also showed reduced physical responses compared to other positions in training, therefore suggesting that 3) TL does not correspond to the match load [24, 25]. 4) Both the metabolic and traditional approaches should be considered together for load monitoring and programming by practitioners; and finally, due to the high-intensity and intermittent nature of soccer, 5) MPev and EDI may be considered relevant variables to represent the physical performance in soccer.

The highest physical responses during the microcycle were found in MD for all positions [10, 26]. These results make sense as ‘players train to compete’ and the main physical objective of training is to withstand the competition demands [1–3], so the need to monitor and programme the TL according to position is well known. In line with other authors, we observed that CMs covered the greatest total distance and high-intensity distance (considering together all speed-range distances > 14 km·h^-1^) as well as DEC events and ACCs (along with FOs) per match [8, 27–32]. The rationale of these high demands may be the increased participation of CMs in both the attacking and defensive phases [33]. Schuth, Carr, Blanes et al. [34] concluded that the limited space available within central areas of the pitch normally occupied by CMs could lead them to potentially perform more hard ACCs and DECs. Therefore, it may suggest that the higher number of ACC and DEC events is due to the specific role of CMs in the game according to the playing style [35]. For instance, if the team loses the ball possession, usually the CMs have to press directly on the opponent with the ball or help the pressing teammate to temporize and avoid counterattacking, while other midfielders retreat by occupying a larger playing area to regain the ball possession [36].

CMs performed the highest measures on MD compared to other positions. However, different patterns were found in training and CMs showed the lowest number of ACCs and DECs every TD. Similarly, FOs also performed the greatest SPD and VHSRD on MD but not among TDs. Considering the variability between playing positions, these results suggest that TL does not correspond to match demands, as it can be logically understood that if CMs cover a greater high-intensity distance in a match, they should perform the longest distance in training. The reason that CMs showed very high variability between training and match loads may be due to a less specific training design for them and coaches used CMs ‘to support’ the specific tasks of other positions, so they were not responsible for performing the decisive ‘final actions’, such as the last sprint of wing players for crossing the ball into the box, or when FOs try to dribble past defenders for penetration into the opponent penalty area [37]. For instance, the common task for ‘crosses and shots to goal’ is more oriented to wing players (EDs and WMs) and FOs, whereas CMs normally act by supporting the exercise with passes or for the second phase. Therefore, it would be recommended to design specific tasks or even specific movements or actions in the tasks that simulate the positional physical demands, in addition to technical-tactical requirements.

CDs and EDs showed similar physical responses in many metrics (e.g. ACCs, DECs, total distance, MP or power events, among others). The mostly similar performance found in defensive positions may be due to the playing style proposed by the team [35], positioning the ‘closed’ defensive line in the central area, thus limiting EDs’ opportunities to attack and then leaving a greater responsibility for WMs to carry out the offensive actions on wings, as well as developing their defensive duties. Nonetheless, the increased values of EDs in the highest intensity metrics (SPD, VHSRD, Pmax and MPev) suggest that they may also be involved in offensive actions on wings, such as counterattacks, in addition to running towards their own goal to defend it. Indeed, several authors grouped all wing players (i.e., EDs and WMs) in the same category as ‘wingers’, due to the similar physical performance developed by them [30, 31]. However, it may be misleading to consider WMs and EDs together, as although they may both show the same physical demands, the type of effort may be opposite (i.e., attacking vs defending). Therefore, it supports the importance of programming an appropriate TL according to the specific demands according to position and playing style [35].

There is conflicting literature analysing the usefulness of using the traditional or metabolic approach to monitor locomotion activity [14, 15, 38, 39]. Most practitioners and scientists use the running speed-based approach to monitor and/or analyse external load as it is considered an easy, validated and well-established method [5, 9, 12]. However, some authors conclude that running-speed metrics (i.e., the traditional approach) do not represent the high-intensity demands of soccer as they do not consider the accelerative phases of high-intensity efforts, thus underestimating the match demands [13–15], whereas the metabolic metrics take into account these activities to calculate the energy cost. However, it should be noted that the metabolic approach does not consider several demanding actions such as collisions, changes of direction or jumps, and the determination of energy expenditure requires consideration of absolute speed and rate of change of speed, as well as time and distance [38]. One objective of this study was to compare metrics from both approaches. In accordance with Castagna et al. [15], who found an almost perfect association between the distance covered at high speed (i.e., ≥ 16 km·h^-1^) and the distance covered at high metabolic power (i.e., ≥ 20 W·kg^-1^), this study also identified positive relationships between metabolic and running speed metrics, suggesting that both approaches may be appropriate for representing high-speed activity in soccer [15]. Likewise, although the metabolic model incorporates both speed and acceleration activity and could provide a more valid measure of energy cost when speed is continuously changing, no current method has yet been accepted as the ‘gold’ standard approach to work rate analysis [38]. In addition, as mentioned above, neither approach considers the non-locomotion metrics such as collision, changes of direction or jumps, and its great impact on physical performance is well known [38]. Nonetheless, several metabolic metrics provide interesting and accurate measures. In this sense, considering that MPev refers to averaged power of bouts considered at ‘high power’ (≥ 20 W·kg^-1^), the decisive actions of a soccer match usually occur at maximal or submaximal intensity [7], and the results showed a significant correlation between MPev and the highest intensity metrics (ACC, DEC, HSRD, VHSRD and SPD), and similar variability between TDs and MD by position, to the authors’ understanding MPev could be considered a reliable measure to represent the players’ physical performance by position. Since MPev is a metric based on an average value and could be difficult to manage for programming the load in daily tasks, this metric could be more useful to consider for accumulated fatigue (i.e., chronic load) than for daily load managing.

EDI may be another interesting metabolic metric, as it represents the ratio of distance covered if total energy is expended at a constant speed to the actual distance covered. The higher the EDI, the more intermittent is the activity [38]. For instance, FOs showed the highest EDI in MD. However, a greater number of DECs was performed by CMs, and both positions showed almost the same number of ACCs. According to the above, EDI is highly related with ACC and DEC events [16, 40], so FOs should show the maximum value in both ACC and DEC metrics. The rationale of this discrepancy is that CMs, in addition to performing a larger number of DECs, also covered a greater total distance, whilst FOs covered a significantly lower total distance. Thus, the FO profile involves more stop–start running [38].

In summary, high workload variability was found among TDs according to position, with higher values and greater positional differences being found in MD compared to training. In match, CMs covered the greatest total distance, high-intensity distance (considering together all speed-ranges distances > 14 km·h^-1^) and greater number of DECs than other positions. However, the load variability found between TDs did not correspond with the match load for all positions (e.g., CMs). Both the metabolic and traditional approaches are appropriate methods for monitoring the training and competition load of professional soccer players. Although most practitioners usually use running speed-based variables for load monitoring, metabolic metrics should also be considered in order to obtain the energy expenditure from the different activities and thus determine the real physical demands of soccer players. In this sense, and due to the intermittent and high-intensity nature of soccer [3, 14], MPev and EDI are interesting metabolic metrics to take into account; MPev represents the energy cost of the activity performed at high power (i.e., high intensity), and EDI reflects the intermittent stop–start running nature of soccer. However, neither approach considers the non-locomotion metrics such as collision, changes of direction or jumps, among others, and it is well known to have a great impact on physical performance.

This study has some limitations. Firstly, the results obtained belong to a single team. Although it is a sample of elite soccer players, the data may not be generalizable to all elite players competing at the same level. Another limitation of this study was not performing individualized load analysis by player, as this information was not available to the authors due to confidentiality issues. Therefore, further research should aim to perform an individualized analysis with a greater number of teams considering different playing styles, and finally the non-locomotion actions should also be included for load monitoring.

## Practical applications

Having good and extensive knowledge about workload monitoring is of paramount importance to practitioners, especially in high-level soccer. Since players train to achieve good performance in competition (i.e., on MD), it is known that programming the load among TDs relative to match load by position is a good way for load monitoring. Both metabolic and traditional running speed-based approaches should be considered together for load monitoring and programming to determine the physical demands of soccer players. In this sense, EDI and MPev seem to be useful metrics to differentiate more clearly the players’ characteristics, taking into account their playing positions. Finally, practitioners should place more emphasis on designing training tasks to attain appropriate TL by position.

## References

[1] Guerrero-Calderón B. The effect of short-term and long-term coronavirus quarantine on physical performance and injury incidence in high-level soccer. Soccer Soc. 2021;22(1–2):85–95.

[2] Malone S, Owen AL, Newton M, Mendes B, Collins KD, Gabbett TJ. The acute: chonic workload ratio in relation to injury risk in professional soccer. J Sci Med Sport. 2017;20(6):561–5. 27856198 10.1016/j.jsams.2016.10.014

[3] Owen AL, Forsyth JJ, Wong DP, Dellal A, Connelly SP, Chamari K. Heart Rate–Based Training Intensity and Its Impact on Injury Incidence Among Elite-Level Professional Soccer Players. J Strength Cond Res. 2015;29(6):1705–12. 26010801 10.1519/JSC.0000000000000810

[4] Gabbett TJ. The training—injury prevention paradox: should athletes be training smarter and harder? Br J Sports Med. 2016;50(5):273–80. 26758673 10.1136/bjsports-2015-095788 PMC4789704

[5] Ehrmann FE, Duncan CS, Sindhusake D, Franzsen WN, Greene DA. GPS and Injury Prevention in Professional Soccer. J strength Cond Res. 2016;30(2):360–7. 26200191 10.1519/JSC.0000000000001093

[6] Guerrero-Calderón B, Klemp M, Morcillo JA, Memmert D. How does the workload applied during the training week and the contextual factors affect the physical responses of professional soccer players in the match? Int J Sports Sci Coach. 2021;174795412199561.

[7] Ade J, Fitzpatrick J, Bradley PS. High-intensity efforts in elite soccer matches and associated movement patterns, technical skills and tactical actions. Information for position-specific training drills. J Sports Sci. 2016;34(24):2205–14. 27537056 10.1080/02640414.2016.1217343

[8] Lago-Peñas C, Rey E, Lago-Ballesteros J, Casáis L, Domínguez E. The Influence of a Congested Calendar on Physical Performance in Elite Soccer. J Strength Cond Res. 2011;25(8):2111–7. 21572352 10.1519/JSC.0b013e3181eccdd2

[9] Owen AL, Lago-Peñas C, Gómez MÁ, Mendes B, Dellal A. Analysis of a training mesocycle and positional quantification in elite European soccer players. Int J Sport Sci Coach. 2017;12(5):665–76.

[10] Malone J, Di Michele R, Morgans R, Burgess D, Morton J, Drust B. Seasonal training-load quantification in elite English premier league soccer players. Int J Sports Physiol Perform. 2015;10(4):489–97. 25393111 10.1123/ijspp.2014-0352

[11] Guerrero-Calderón B, Klemp M, Castillo-Rodriguez A, Morcillo JA, Memmert D. A New Approach for Training-load Quantification in Elite-level Soccer: Contextual Factors. Int J Sports Med. 2020;41:1–9. 10.1055/a-1289-9059 33321524

[12] Malone J, Lovell R, Varley MC, Coutts AJ. Unpacking the Black Box: Applications and Considerations for Using GPS Devices in Sport. Int J Sports Physiol Perform. 2017;12(Suppl 2):S2-18–S2-26. 10.1123/ijspp.2016-0236 27736244

[13] Gaudino P, Iaia FM, Alberti G, Hawkins RD, Strudwick AJ, Gregson W. Systematic bias between running speed and metabolic power data in elite soccer players: Influence of drill type. Int J Sports Med. 2014;35(6):489–93. 24165959 10.1055/s-0033-1355418

[14] Gaudino P, Iaia FM, Alberti G, Strudwick AJ, Atkinson G, Gregson W. Monitoring training in elite soccer players: systematic bias between running speed and metabolic power data. Int J Sports Med. 2013;34(11):963–8. 23549691 10.1055/s-0033-1337943

[15] Castagna C, Varley M, Póvoas SCA, D’Ottavio S. Evaluation of the Match External Load in Soccer: Methods Comparison. Int J Sports Physiol Perform. 2017;12(4):490–5. 27618733 10.1123/ijspp.2016-0160

[16] Polglaze T, Hoppe MW. Metabolic Power: A Step in the Right Direction for Team Sports. Int J Sports Physiol Perform. 2019;14(3):407–11. 30732493 10.1123/ijspp.2018-0661

[17] Osgnach C, Di Prampero PE. Metabolic Power in Team Sports – Part 2: Aerobic and Anaerobic Energy Yields. Int J Sports Med. 2018;39(08):588–95. 29902809 10.1055/a-0592-7219

[18] Martín-López Á, Mendes RS, Castillo-Rodríguez A. Internal and External Loads in Training Week Before the Competition in U19 High-Level Soccer Players. J strength Cond Res. 2018;Publish Ah(00):1–7. 30557176 10.1519/JSC.0000000000002975

[19] Owen AL, Dunlop G, Rouissi M, Haddad M, Mendes B, Chamari K. Analysis of positional training loads (ratings of perceived exertion) during various-sided games in European professional soccer players. Int J Sport Sci Coach. 2016;11(3):374–81.

[20] Hoppe MW, Baumgart C, Slomka M, Polglaze T, Freiwald J. Variability of Metabolic Power Data in Elite Soccer Players during Pre-Season Matches. J Hum Kinet. 2017;58(1):233–45. 28828094 10.1515/hukin-2017-0083 PMC5548171

[21] Tomczak M, Tomczak E. The need to report effect size estimates revisited. An overview of some recommended measures of effect size. Trends Sport Sci. 2014;1(21):19–25.

[22] Coe R, Merino C. Magnitud del efecto: Una guía para investigadores y usuarios. Rev Psicol a – PUCP. 2003;21(1):147–77.

[23] Hopkins WG, Marshall SW, Batterham AM, Hanin J. Progressive statistics for studies in sports medicine and exercise science. Med Sci Sports Exerc. 2009;41(1):3–12. 19092709 10.1249/MSS.0b013e31818cb278

[24] Enes A, Oneda G, Alves DL, Palumbo D de P, Cruz R, Moiano Junior JVM, et al. Determinant Factors of the Match-Based Internal Load in Elite Soccer Players. Res Q Exerc Sport. 2021;92(1):63–70. 32027579 10.1080/02701367.2019.1710445

[25] Brito J, Hertzog M, Nassis GP. Do Match-Related Contextual Variables Influence Training Load in Highly Trained Soccer Players? J Strength Cond Res. 2016;30(2):393–9. 26244827 10.1519/JSC.0000000000001113

[26] Martín-García A, Gómez Díaz A, Bradley PS, Morera F, Casamichana D. Quantification of a professional football team’s external load using a microcycle structure. J Strength Cond Res. 2018;32(12):3511–8. 30199452 10.1519/JSC.0000000000002816

[27] Abt G, Lovell R. The use of individualized speed and intensity thresholds for determining the distance run at high-intensity in professional soccer. J Sports Sci. 2009;27(9):893–8. 19629838 10.1080/02640410902998239

[28] Al Haddad H, Simpson BM, Buchheit M, Di Salvo V, Mendez-Villanueva A. Peak match speed and maximal sprinting speed in young soccer players: Effect of age and playing position. Int J Sports Physiol Perform. 2015;10(7):888–96. 25710125 10.1123/ijspp.2014-0539

[29] Di Salvo V, Gregson W, Atkinson G, Tordoff P, Drust B. Analysis of High Intensity Activity in Premier League Soccer. Int J Sports Med. 2009;30(03):205–12. 19214939 10.1055/s-0028-1105950

[30] Mohr M, Krustrup P, Bangsbo J. Match performance of high-standard soccer players with special reference to development of fatigue. J Sports Sci. 2003;21(7):519–28. 12848386 10.1080/0264041031000071182

[31] Rampinini E, Coutts AJ, Castagna C, Sassi R, Impellizzeri F. Variation in Top Level Soccer Match Performance. Int J Sports Med. 2007;28(12):1018–24. 17497575 10.1055/s-2007-965158

[32] Rampinini E, Impellizzeri FM, Castagna C, Coutts AJ, Wisløff U. Technical performance during soccer matches of the Italian Serie A league: Effect of fatigue and competitive level. J Sci Med Sport. 2009;12(1):227–33. 18083631 10.1016/j.jsams.2007.10.002

[33] Silva B, Garganta J, Santos R, Teoldo I. Comparing tactical behaviour of soccer players in 3 vs. 3 and 6 vs. 6 small-sided games. J Hum Kinet. 2014;41(1):191–202. 25114746 10.2478/hukin-2014-0047 PMC4120453

[34] Schuth G, Carr G, Barnes C, Carling C, Bradley PS. Positional interchanges influence the physical and technical match performance variables of elite soccer players. J Sports Sci. 2016;34(6):501–8. 26700131 10.1080/02640414.2015.1127402

[35] Tierney PJ, Young A, Clarke ND, Duncan MJ. Match play demands of 11 versus 11 professional football using Global Positioning System tracking:Variations across common playing formations. Hum Mov Sci. 2016;49:1–8. 27269201 10.1016/j.humov.2016.05.007

[36] Castelão D, Garganta J, Santos R, Teoldo I. Comparison of tactical behaviour and performance of youth soccer players in 3v3 and 5v5 small-sided games. Int J Perform Anal Sport. 2014;14(3):801–13.

[37] Lago-Ballesteros J, Lago-Peñas C, Rey E. The effect of playing tactics and situational variables on achieving score-box possessions in a professional soccer team. J Sports Sci. 2012;30(14):1455–61. 22856388 10.1080/02640414.2012.712715

[38] Polglaze T, Dawson B, Peeling P. Gold Standard or Fool’s Gold? The Efficacy of Displacement Variables as Indicators of Energy Expenditure in Team Sports. Sport Med. 2016;46(5):657–70. 10.1007/s40279-015-0449-x 26643522

[39] Martínez-Cabrera FI, Núñez-Sánchez FJ. The use of metabolic power to assess physical demands in soccer: How does it differ from the traditional approach through speed running? J Sports Med Phys Fitness. 2018;58(10):1403–11. 28745474 10.23736/S0022-4707.17.07563-6

[40] Osgnach C, Poser S, Bernardini R, Rinaldo R, Di Prampero PE. Energy cost and metabolic power in elite soccer: A new match analysis approach. Med Sci Sports Exerc. 2010;42(1):170–8. 20010116 10.1249/MSS.0b013e3181ae5cfd

